# The role of microtransactions in Internet Gaming Disorder and Gambling Disorder: A preregistered systematic review

**DOI:** 10.1016/j.abrep.2022.100415

**Published:** 2022-02-22

**Authors:** Phillip C. Raneri, Christian Montag, Dmitri Rozgonjuk, Jason Satel, Halley M. Pontes

**Affiliations:** a School of Psychological Sciences, University of Tasmania, Churchill Avenue, Hobart, TAS, Australia; b Department of Molecular Psychology, Institute of Psychology and Education, Ulm University, Ulm, Germany; c Institute of Mathematics and Statistics, University of Tartu, Tartu, Estonia; d Department of Organizational Psychology, Birkbeck, University of London, London, United Kingdom

**Keywords:** Microtransaction, Loot box, Gaming, Gambling, Systematic review

## Abstract

Recently there has been increased interest in understanding the relationship between microtransactions, gaming, and gambling. This review aimed to synthesise the evidence on the relationship between microtransactions, ‘Internet Gaming Disorder’ (IGD), and Gambling Disorder in order to report on the: psychometric assessments used, sampling and demographic information, study design and sampling methods, relationships between microtransactions and both IGD and gambling disorder. Inclusion criteria included: refereed studies quantifying microtransactions and/or loot boxes examining their relationship with IGD and/or gambling disorder that were published between 2013 and 2021. Electronic databases were searched and the results were synthesised qualitatively. 14 studies were included. The quality of the evidence was ‘Good’ and clear positive relationships between microtransactions and both IGD and gambling disorder were identified. These relationships apply more to loot boxes than other microtransactions, and risky loot box use was identified as a possible mediator of these relationships. Additionally, microtransaction expenditure increased with the risk of gambling disorder. There is some evidence that adolescents who purchase loot boxes may be more at risk of developing gambling disorder. External validity is limited due to the cross-sectional nature of the evidence, the use of convenience sampling, and the predominantly Western samples resulting in non-representative samples. Prevalence rates of IGD and gambling disorder varied significantly across studies and were different to general prevalence rates. We conclude that there is a need to develop consistent methods for assessing IGD and microtransaction engagement in future research. Implications for policy-makers and future research are discussed.

## Highlights

• Microtransaction engagement is associated with gaming and gambling disorder.
• Loot boxes appear to pose greater risk for addiction than other microtransactions.
• Greater in-game expenditure increased with risk of gambling disorder.
• Prevalence rates of gaming and gambling disorder varied significantly.

## 1 Introduction

Electronic gaming has become extremely prevalent in the modern world. According to recent large-scale studies, around 65% of American adults and 66% of Australians play video games, with 90% of Australian households having at least one device dedicated to playing video games (Brand et al., 2019, Entertainment Software Association, 2019). In reference to key demographics using convenience samples, in Australia alone 69% of people aged 1–17 years old, 62% of people aged 18–64 years old, and 42% of people aged 65–94 played video games. The average gamer was aged 33–34 years old and 47% of gamers were female (Brand et al., 2019). According to Newzoo (2020), the global gaming market had $USD159.3 billion in revenue in 2020, and it is estimated that in 2023 there will be 3.07 billion gamers in the world. These statistics illustrate that gaming is a pervasive activity that is enjoyed throughout the lifespan and not only by men, further moving away from the stereotypical image of gamers being lonely teenage boys (Pontes, 2018). As electronic gaming is a relatively new phenomenon with such prevalent participation, it is imperative to investigate the impacts it has on the lives of all individuals worldwide.

Accompanying the popularity of electronic gaming is a wide range of associated positive and negative psychosocial outcomes (Pontes, 2018, Saunders et al., 2017). At the qualitative level, gamers in Australia report that playing any sort of video game helps them reduce stress, keeps their mind active, and helps with not only creativity, but also emotional, and social wellbeing (Brand et al., 2019). Additionally, playing shooter games at moderate levels has been found to result in a variety of cognitive benefits such as faster and more accurate allocation of attention, better spatial resolution in visual processing, and improved spatial skills (Granic et al., 2014, Howard et al., 2017). Nevertheless, null effects in relation to these cognitive benefits have been reported in recent research (Sala, Tatlidil, & Gobet, 2018). For a more complete review on the benefits of playing video games see Griffiths (2019).

Furthermore, a recent systematic review and meta-analysis on the relationship between the Big Five personality factors and gaming (Akbari et al., 2021) revealed that most personality factors (i.e., agreeableness, openness to new experiences, and extraversion) may be somewhat protective of engaging in problematic gaming behaviours. Neuroticism was found to have either a positive relationship with problem gaming or no relationship at all. Conscientiousness was found to be the most protective against problem gaming. This was attributed to conscientious individuals being more likely to be organised and prioritise their ‘real life’ goals, and therefore have a healthier relationship with gaming.

Conversely, excessive gaming has been shown to be associated with psychiatric disorders (e.g., depression and anxiety) alongside addictive and aggressive behaviours (Greitemeyer, 2018, Pontes, 2018, Wang et al., 2018), with a recent large-scale study (Pontes, Schivinski, Kannen, & Montag, 2022) including 123,262 gamers reporting that disordered gaming translates to an average of 34.53 to 40.13 hours of weekly time spent gaming. Additionally, Akbari et al. (2021) found that neuroticism, in some cases, is a personality vulnerability factor that may result in an individual developing problematic gaming behaviours. It was thought that this may be due to the fact that neurotic individuals are less confident, and may use gaming as a way to cope with, suppress, or escape negative emotions. A recent large scale study by Montag, Schivinski, & Pontes, (2021) supports the already mentioned findings that high neuroticism and low conscientiousness seem to be the driving personality factors of gaming disorder tendencies.

Evidence of disordered gaming first came to light in the 1980s (Klein, 1984, Nilles, 1982, Ross et al., 1982, Soper and Miller, 1983) and its status as a mental health disorder has been heavily debated by scholars (Billieux et al., 2015, Griffiths et al., 2016, Kuss, 2013, Maraz et al., 2015). This debate intensified after ‘Internet Gaming Disorder’ (IGD) was included in Section III of the *Diagnostic and Statistical Manual of Mental Disorders,* Fifth edition (DSM-5; American Psychiatric Association, 2013) as a condition for further study.

According to the DSM-5, IGD is defined as persistent and recurring internet use to play games, usually with other people, which results in clinically significant impairment or distress as indicated by at least five of the following nine criteria within a 12-month period: (1) preoccupation with video games, (2) withdrawal symptoms in the absence of gaming, (3) tolerance as indicated by increasing amounts of time spent engaging in gaming, (4) unsuccessful attempts to control participation in gaming, (5) loss of interest in previous hobbies/entertainment due to (and with the exception of) gaming, (6) continued excessive use of video games despite knowledge of psychosocial problems, (7) deceiving family members, therapists, or others about the amount of gaming undertaken, (8) use of video games in order to escape or relieve negative moods, and (9) jeopardising or losing a significant relationship, job, or educational or career opportunity because of participation in video games.

Following the inclusion of IGD in Section III of the DSM-5, the World Health Organization (WHO) recognised ‘Gaming Disorder’ (GD, 6C51) as an official diagnosis in the *International Classification of Disease*, 11th edition (ICD-11; World Health Organization, 2019). According to the ICD-11, GD is classified by a pattern of recurrent and persistent gaming behaviour (online and/or offline), as indicated by the following three criteria: (1) impaired control over gaming, (2) increasing priority assigned to gaming to the point that it takes priority over daily activities and life interests, and (3) continued/increased gaming despite negative consequences. Such dysregulated gaming behaviours must result in significant impairment in personal, family, social, educational, occupational, or other important areas of functioning in order to be classified as GD.

A recent review study (Stevens, Dorstyn, Delfabbro, & King, 2021) reported that the worldwide prevalence of IGD ranged from 1.96% to 3.05%, although significant variance in prevalence rates was found among published studies. In terms of gender differences, the prevalence rates for males ranged from .23% to 22.7%, while for females it ranged from .04% to 9.32%. Although IGD prevalence rates vary significantly, it is apparent that IGD is a prominent phenomenon worldwide, and is thus paramount to be further investigated as it may be affecting a relatively large number of individuals, both directly and indirectly.

The inclusion of GD as a bona fide disorder in the ICD-11 resulted in a new era of research (Pontes & Griffiths, 2020), enabling future research to consistently investigate the aetiology, epidemiology, clinical features, comorbidities, and negative impacts of this disorder. Throughout this review both IGD and GD will be referred to as IGD as most of the studies conducted in the field still adopts the APA’s framework and nomenclature to describe disordered gaming.

One crucial emerging area of research focuses on the relationship between IGD and Gambling Disorder. The similarity between gaming and gambling is not a novel realisation, with the structural and potential addictive similarities being brought to light in the 1990s (Griffiths, 1991). However, with the advancement of technology, the gambling and gaming environments have changed and research on arcade games and slot machines may no longer be relevant in a modern-day context (Macey & Hamari, 2018).

Not only have gaming and gambling changed drastically over the years, but these two activities became intrinsically intertwined (Rozgonjuk, Schivinski, Pontes, & Montag, 2021). Gambling-like mechanisms are being introduced into a broad range of video games (Macey & Hamari, 2018). For example, at The Diamond Casino and Resort in the video game ‘Grand Theft Auto V’ gamers can use real money to gamble (Rockstar Games, 2013), and internet gambling is becoming a more prominent and attractive feature in social media and video games (King, Delfabbro, Kaptsis, & Zwaans, 2014). The nature of gaming also has been changed with the advent of smartphones, allowing mobile access to video games from anywhere, while also giving rise to new games such as ‘Freemium’ games (i.e., Free and Premium; see also Montag, Lachmann, Herrlich, & Zweig, 2019). Although, Freemium games are ‘free to play’, they offer in-game purchases for real money (e.g., currency, virtual goods, or ‘skins’), which are referred to as ‘microtransactions’ (King & Delfabbro, 2018). Therefore, the ‘Premium’ in these Freemium games need to be paid for.

Furthermore, microtransactions can take many different forms, such as one-off specific purchases, timed or permanent (e.g., item is for sale for 24 hours or item is always for sale), or can alternatively be in the form of random purchases known as ‘loot boxes’. Some microtransactions are available in video games that do not require real money, however, microtransactions utilising real currency have become a dominant business model within the video game industry (Tomić, 2017). Loot boxes, when opened, provide in-game rewards on a chance basis, and are purchased with the hope of collecting a rare or valuable reward that is worth more than the cost of the loot box (Yokomitsu, Irie, Shinkawa, & Tanaka, 2021). In some games, loot boxes require ‘keys’ to open them, and, in some cases, gamers are able to earn both keys and loot boxes without using real money (Yokomitsu et al., 2021). However, they are often purchased using real-world currency. Both loot boxes and gambling can rely on an individual risking money on the outcome of a chance event with the goal of receiving a reward of high value (Zendle & Cairns, 2018). Opening loot boxes is often accompanied by exciting effects such as sounds and lights (McCaffrey, 2019), which is similar to what occurs in a live ‘brick and mortar’ gambling context (i.e., sound effects, music, and lights being triggered when you win on a slot machine). Gambling rewards are also similar to rewards obtained by opening loot boxes, as intermittent reinforcement plays an important role. Not all loot boxes will provide a valuable item (i.e., win) every time, but only from time to time, similarly to gambling rewards.

### 1.1 Rationale for the current review

The similarity between loot boxes and gambling has led to concern that loot box purchases may lead to or exacerbate gambling disorder symptoms (Zendle & Cairns, 2018). Gambling disorder can be defined as a pattern of excessive gambling behaviours which causes problems for an individual in their personal, vocational, and/or family life (Raylu and Oei, 2004, Zendle and Cairns, 2018). This is thought to occur when an individual becomes conditioned to the arousing features of gambling so much so that the need to be excited through gambling causes significant harm to the self and others (Blaszczynski & Nower, 2002). Interestingly, the WHO’s criteria for Gambling Disorder (6C50) are nearly the same as for GD, but clearly the terms “gaming” and “gambling” need to be exchanged. It is possible that conditioning can occur to a similar effect through the use of loot boxes, which could lead to an increase in gambling disorder amongst those with IGD who engage in loot box purchasing, especially risky loot box purchasing (Zendle & Cairns, 2018). Risky loot box purchasing refers to purchasing behaviours that have typical addictive motivations, such as opening loot boxes for the ‘thrill’ of it (but see overlap with impulse control disorders where thrill in parts plays also a role), playing video games for longer than intended in order to earn loot boxes, and putting off other activities in order to obtain a loot box (Brooks & Clark, 2019).

Research into the relationship between IGD and gambling disorder within the context of microtransactions is still in its infancy. However, understanding these interactions is imperative for policy-makers to inform and guide policy surrounding young people, gaming, and gambling, as well as for clinicians to guide treatment, intervention, and develop effective harm minimisation strategies.

At the time this project was initiated, no systematic review study had been conducted to investigate the relationship between IGD and gambling disorder in the context of microtransactions. However, at the time of writing of this study there were at least three review studies that have been published as of September 2021 (see Garea et al., 2021, Spicer et al., 2021, Yokomitsu et al., 2021). Despite this, the present review is still warranted as it builds and expands upon the previously published reviews in the field since these previous reviews have focused on a specific type of microtransaction, such as loot boxes (Garea et al., 2021, Spicer et al., 2021, Yokomitsu et al., 2021). Moreover, the present review included a slightly different set of studies compared to the previously published reviews, thus, generating new insights into the problem under investigation. These previously published reviews all found a significant relationship between loot boxes and IGD and gambling disorder. However, it is important to examine the relationship between both loot boxes and other non-random microtransactions with IGD and gambling disorder in order to further understand whether the gambling-like structure of loot boxes plays a unique role in these relationships.

### 1.2 Objectives

The current review aims to synthesise and evaluate the literature on microtransactions and their relationships with both IGD and gambling disorder. To achieve this goal, this study will synthesise and evaluate the existing literature in order to report on the following methodological and psychological features: **Methodological features*:*** (1) measurement and psychometric assessments typically adopted, (2) sample characteristics and demographics information, and (3) study design and sampling characteristics. **Psychological features:** (4) relationship between microtransactions and gambling disorder, (5) relationships between microtransactions and IGD, and the (6) associations between microtransactions, IGD, and gambling disorder. Doing so will provide insight into the potential harm microtransactions may have on individuals and inform policy-makers on how to better protect vulnerable people from harms associated with microtransaction engagement.

## 2 Methods

The current review methodology, including the research question, search strategy, inclusion and exclusion criteria, and risk of bias assessments were developed a priori and were described in the preregistration protocol (PROSPERO CRD42020216371, https://www.crd.york.ac.uk/prospero/display_record.php?ID=CRD42020216371; Raneri & Pontes, 2020). The present review was conducted in accordance with the latest revised guidelines for the Preferred Reporting Items for Systematic Reviews and Meta-Analyses (PRISMA) checklist (Page et al., 2021, Page et al., 2021).

Additionally, the Risk of Bias in Systematic Reviews tool (ROBIS; Whiting et al., 2016) was used to assess the risk of bias within the present review, which was completed by the first author in consultation with the last author. ROBIS consists of four primary domains where bias can be introduced in a systematic review: (1) the eligibility criteria, (2) the identification and selection of studies, (3) data collection and study appraisal, and (4) the review’s synthesis and findings. For each domain the rater was required to answer five to six questions regarding the review process and answer ‘Yes’, ‘Probably Yes’, ‘Probably No’, ‘No’, or ‘No Information’. ‘Yes’ and ‘Probably Yes’ answers indicate low levels of bias in the review, ‘No’ and ‘Probably No’ indicate higher levels of bias in the review, and ‘No Information’ responses indicate unclear levels of bias. Each domain was then rated as ‘Low Risk’, ‘High Risk’ or ‘Unclear’, and the rater was required to record any rationale for concerns. The rater was then asked whether any concerns in domains 1–4 were addressed in the study, if the included studies were appropriate to the research question, and if the researchers avoided emphasising results based on their statistical significance. An overall rating of ‘low’ ‘high’ or ‘unclear’ risk of bias is given to the review based on these final questions.

### 2.1 Inclusion criteria

A set of strict inclusion criteria was adopted when deciding if a study should be included in the review or not. Each study had to be a peer-reviewed empirical investigation that operationalised microtransactions and/or loot boxes and examined their relationship with either IGD, gambling disorder, or both IGD and gambling disorder. Only studies published in English, Spanish, or Portuguese in refereed journals were included as these were the languages spoken between the reviewing authors. Studies required a publication date between 2013-2021. The cut-off date of 2013 was used as it was the year that IGD was first formally and tentatively introduced as a potential behavioural addiction in the DSM-5 (American Psychiatric Association, 2013), which encouraged and enabled further research to be carried out into the aetiology and epidemiology of the disorder. Studies were excluded from the review if they were theoretical or qualitative studies, single case studies (i.e., studies in which *N* = 1), or did not meet the above inclusion criteria.

### 2.2 Search strategy and screening process

PsycARTICLES, PsycINFO, PubMed, Scopus, and Web of Science were searched on 15/09/2020 using the following search strategy: (microtransac* OR loot box* OR purchas*) AND ((disorder* OR patholog* OR problem* OR addict* OR compulsive OR dependen*) AND (video OR computer OR internet OR gaming OR game OR gamb*)). The development of the search strategy was based on previous literature and was assisted by an independent academic librarian from the University of Tasmania. Different search strategies were piloted and the strategy that yielded the highest number of relevant studies was utilised in the final search. The search strategy was adapted for use with each individual database and only titles and abstracts were searched.

The search results were imported into a systematic review management website (i.e., Covidence; Veritas Health Innovation, n.d.) to remove duplicates. Next, titles and abstracts were screened by the first author and potentially eligible studies were collated using Microsoft Excel (Microsoft Corporation, n.d.). The last author then reviewed the potentially eligible studies and discrepancies were discussed between the research team. The first author then reviewed the full texts based on the inclusion and exclusion criteria outlined. The last author then reviewed the full texts independently, with emerging discrepancies being discussed and resolved within the team via an online discussion aimed at resolving these discrepancies by reaching a joint decision. Inter-rater agreement coefficients were calculated using Fleiss’ Kappa analysis to assess the level of agreement between raters (coded as ‘*1 = study to be included*’ and ‘*0 = study not to be included*’) in relation to the final inclusion of eligible studies. The results of this analysis provided coefficients between .61 and .80, which indicate substantial agreement among raters (Landis & Koch, 1977). Publications were then separated into three groups based on studies that investigated (i) microtransactions and gambling disorder, (ii) microtransactions and IGD, or (iii) microtransactions and both IGD and gambling disorder.

An Excel spreadsheet was used to extract the following data from eligible studies: design of the study, participant demographics, prevalence rates of IGD and gambling disorder, the types of microtransactions measured, microtransaction expenditure, correlation coefficients, regression coefficients, mean difference estimates, effect sizes, and psychometric tests utilised. If the data set of a study was made available online, it was examined to extract data that was measured but not reported in the published report. When necessary, corresponding authors of the eligible studies were directly contacted via email to request additional data that was not publicly available. Seven authors were sent emails regarding nine studies and additional information was provided from five authors regarding their respective five studies. One author was unable to provide additional information while another author did not respond despite a follow up email. For transparency purposes, the final data set for the present study is publicly available at the Open Science Framework (see Raneri & Pontes, 2020, https://osf.io/9gepb/).^1^

### 2.3 Quality appraisal

The Appraisal tool for Cross-Sectional Studies (AXIS; Downes, Brennan, Williams, & Dean, 2016) was used to assess the quality of the evidence and risk of bias in the studies included in the present review. This deviated from the preregistered plan. Originally, the Grading of Recommendations Assessments, Development and Evaluation (GRADE; Schünemann, Brożek, Guyatt, & Oxman, 2013) tool was going to be used to assess evidence quality. However, it was later deemed that the GRADE tool was more suited for assessing evidence quality for intervention studies. As such, the AXIS tool was identified after preregistration and was considered to be more appropriate as none of the included studies were intervention studies. Additionally, almost all of the journal articles identified in the search performed were cross-sectional, for which the AXIS tool is specifically designed to assess. One study by Zendle (2019) was a prospective cohort study; however, the questions were still appropriate for assessing the quality of the evidence in that study.

The AXIS tool has 20 different questions for each journal article that are answered with ‘Yes’, ‘No’, or “Maybe’. The wording for question 13 was shifted slightly for ease of reporting and consistency with the other questions so that all ‘Yes’ answers indicated good quality of evidence or low risk of bias and ‘No’ answers indicated poor quality of evidence or high risk of bias. Questions 13 and 14 were often marked non applicable as response rate and information about non responders were not measurable due to the nature of the study.

The studies included in this review were evaluated based on these 20 questions following the AXIS guidelines. A percentage score was calculated based on the number of questions answered as ‘Yes’ divided by the total number of applicable questions for each journal article. The methodological quality of each study was categorised into one of four categories: poor (0–24%), fair (25–49%), good (50–74%) or excellent (75–100%) based on previous studies (Maass et al., 2015, Yokomitsu et al., 2021). The quality appraisal was conducted by the first author in consultation with the last author who helped resolve outstanding concerns about the rating of the studies.

## 3 Results

### 3.1 Literature search and selection process

Brooks and Clark's (2019) article reported the findings of two separate studies (in terms of samples). For the purpose of this review, the article was included as two separate studies which were then reviewed and reported as such. The initial searches performed for the literature review yielded 752 articles. Duplicate articles were removed (*n* = 187), which left 565 entries to be screened. After screening titles and abstracts, 549 articles were removed, leaving 16 articles for full-text review. One additional study (Hall, Drummond, Sauer, & Ferguson, 2021) was published after the search was conducted and identified during the data extraction phase as being a potential study for inclusion. This study was also included resulting in a total of 17 articles for full-text review.

After reviewing the full-texts, three articles were excluded due to not meeting the inclusion criteria: One study (Columb, Griffiths, & O'Gara, 2020) was further excluded as it did not include inferential statistics, another study (Macey & Hamari, 2018) was excluded as it did not clearly operationalise microtransaction and loot box expenditure, and one final study (Hall et al., 2021) was excluded as it only investigated the variables within the context of the COVID-19 pandemic and did not have baseline data that could be compared to the other studies.

**Fig. 1:**
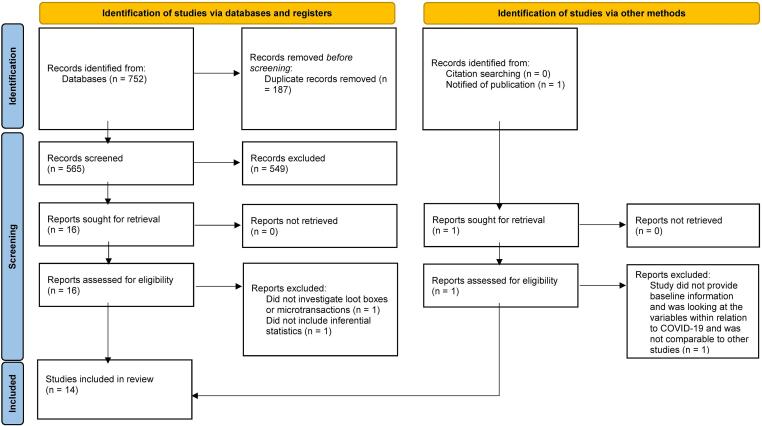
Prisma Flow chart.

As a result, a total of 14 studies from 13 publications were eligible for inclusion in the review (Brooks and Clark, 2019, Drummond et al., 2020, A. King et al., 2020, D. King et al., 2020, Kristiansen and Severin, 2020, Li et al., 2019, Macey and Hamari, 2019, Zendle, 2019, Zendle, 2020, Zendle and Cairns, 2018, Zendle and Cairns, 2019, Zendle et al., 2020, Zendle et al., 2019). The results of the Fleiss’ Kappa analysis yielded: *κ* = .63, *z* = 2.44, *p* = .015, which indicates substantial agreement between authors when reviewing full-texts for inclusion in the final review (Landis & Koch, 1977). The reference lists of these studies were searched for additional studies to include; however, none were identified. See Fig. 1 for PRISMA flow diagram. The ROBIS assessment identified a low risk of bias within this review when considering study eligibility criteria, study identification and selection, data collection and appraisal, and synthesis of findings.

### 3.2 Sample characteristics and demographic information

See Table 1 for sample and demographic information. The sample sizes ranged from N = 113 to N = 7,422. In terms of gender proportions, nine studies (Brooks and Clark, 2019, D. King et al., 2020, Li et al., 2019, Macey and Hamari, 2019, Zendle, 2019, Zendle and Cairns, 2018, Zendle and Cairns, 2019, Zendle et al., 2020, Zendle et al., 2019) had more male participants than females (i.e., >60% males), one study (Drummond et al., 2020) had more females than males (i.e., >60% females), and four studies (Brooks and Clark, 2019, A. King et al., 2020, Kristiansen and Severin, 2020, Zendle, 2020) had equal distributions of males and females. The proportion of male participants ranged from 35.46% to 91.92% while the proportion of female participants ranged from 5.50% to 63.39%, and the proportion of non-binary, other, or non-specified genders ranged from .56% to 4.46%.

**Table 1:** Sample and demographic information.

Study	Sample Size	Sex/Gender % (*n*)			Ethnicity/Nationality %	Age (Mean, SD)	Targeted/Type Participants	Represen-tative
		M	F	NB/O/Not Specified				
Brooks and Clark, (2019)†	144	51.39% (74)	48.61% (70)	N/A	Asian = 8.32% African American/Black = 8.33% Caucasian/white = 78.50% Latin American = 1.40%, Other = 3.47%	Median (SD) 34.00 (10.00)	North American adults (≥21 years), general population	No
Brooks and Clark, (2019)†	113	87.61% (99)	12.39% (14)	N/A	Asian = 62.10% African American/Black = .90% Caucasian/white = 24.10% Latin American = .90% Other = 12.00%	Median (SD) 21.00 (2.39)	European/British emerging adults (≥19 years), students	No
Drummond et al. (2020)	1049	35.46% (372)	63.39% (665)	1.14% (12)	Australia = 32.32% New Zealand = 30.79 USA = 36.89%	38.08 (14.58)	New Zealand, Australian, and American, general population	No
King, Wong-Padoongpatt et al. (2020)	263	49.05% (129)	50.95% (134)	N/A	Asian = 5.20% Black = 21.30% Latinx = 10.50, White = 64.80 Other = 4.50	22.79 (2.00)	Adults, 18–25, in the US who are not students, and understand written English.	No
	428	91.82% (393)	6.54% (28)	1.64% (7)	US Nationals = 48.10% Australian = 7% Canadian = 6.50% UK = 5.80%	23.50 (7.30)	Adults (≥18 years) who play Fortnite	No
Kristiansen and Severin (2020)	1137	49.42% (562)	50.57% (575)	N/A	N/A	N/A (Ordinal Data)	Danish adolescents who play games on PC or video game console (12–16 years, n = 995)	Yes
Li et al. (2019)	618	63.75% (394)	36.24% (224)	N/A	N/A	27 (8.90)	Adult (≥18 video) gamers	No
Macey and Hamari (2019)	582	91.92% (535)	5.50% (32)	.69% (4)	American = 35.57% Australian = 3.09% British = 7.9% Canadian = 6.7% Finnish = 7.04% German = 4.64% Other = 33.16%	N/A (Ordinal Data)	English speaking video gamers who had watched esports, gambles, or purchased loot boxes within 12 m	No
Zendle (2019)	112	71.43% (80)	24.11% (27)	4.46% (5)	N/A	N/A (Ordinal Data)	Adults (≥18 years) who play Heroes of the Storm	No
Zendle (2020)	1081	48.65% (526)	50.79% (549)	.56% (6)	White = 80.66% mixed = 2.68% Black = 3.52% Asian = 7.03% Other = 1.11%	N/A (Ordinal Data)	UK Adults (≥18 years), general population	Yes
Zendle and Cairns (2018)	7422	89.09% (6612)	8.74% (649)	N/A (2.17% (161))	USA = 44% UK = 8% Canada = 7% NR = 5% 'There were respondents from 92 other countries’	N/A (Ordinal Data)	Adult (≥18 years) gamers	No
Zendle and Cairns (2019)	1172	64.07% (751)	31.74% (372)	4.27% (50)	USA = 100%	N/A (Ordinal Data)	Adult (≥18) American gamers	No
Zendle et al. (2020)	1200	60.75% (729)	37.08% (445)	N/A (2.17% (26))	N/A	N/A (Ordinal Data)	Adults (≥18 years) who were involved in LBs in last month	No
Zendle et al. (2019)	1155	88.31% (1020)	9.26% (107)	2.42% (28)	N/A	17.21 (.83)	Older adolescent gamers 16–18	No

SD = Standard Deviation. NR = Not Reported. LB = Loot Box. M = Male. F = Female. NB = Non-binary. *n* = number of participants in that group. All studies with more than two authors have been written as ‘et al.’. All values have been rounded to 2 decimal places. **†**: Both entries pertain to the same publication, however they report to two separate studies.

Age was reported as an ordinal variable across seven of the 14 studies (Kristiansen and Severin, 2020, Macey and Hamari, 2019, Zendle, 2019, Zendle, 2020, Zendle and Cairns, 2018, Zendle and Cairns, 2019, Zendle et al., 2020) and the categorisation of age was not consistent across these studies, thus, restricting the extraction and comparison of age-related demographic information for these studies. For example, Macey and Hamari (2019) categorised age into groups with a range of 3 (e.g., 15–17 years, 18–21 years, and so on) whereas Zendle (2019) categorised age into groups with a range of seven (e.g., 18–24 years) or five (e.g., 25–29 years, 30–34 years, and so on). Brooks and Clark (2019) reported the median age for both their studies, and the mean was used for all other studies.

The mean and median age of participants ranged from 17.21 to 38.08 years, with standard deviations ranging from .83 to 14.58. Moreover, a total of 10 studies only included adults in their sample (Brooks and Clark, 2019, A. King et al., 2020, D. King et al., 2020, Li et al., 2019, Zendle, 2019, Zendle, 2020, Zendle and Cairns, 2018, Zendle and Cairns, 2019, Zendle et al., 2020) and the age of what constituted an adult varied between studies. One study (Kristiansen & Severin, 2020) investigated adolescents between the ages of 12–16 years, and another study (Zendle et al., 2019) investigated older adolescents between the ages of 16 and 18 years. Two studies did not exclude participants based on age (Drummond et al., 2020, Macey and Hamari, 2019).

Ethnicity and nationality were reported in nine of the eligible studies (Brooks and Clark, 2019, Drummond et al., 2020, A. King et al., 2020, D. King et al., 2020, Macey and Hamari, 2019, Zendle, 2020, Zendle and Cairns, 2018, Zendle and Cairns, 2019), however, there was no consistency in how it was measured, rendering comparisons inviable. Overall, most studies had ‘Caucasian’ dominant samples, except for one study (Brooks & Clark, 2019) which had a majority of participants who described themselves as ‘Asian’ (62.1%). Two studies included samples that were considered representative of the general population (Kristiansen and Severin, 2020, Zendle, 2020).

### 3.3 Study design and sampling methods

All studies included in this review were cross-sectional in design, except for one study (Zendle, 2019) which was a prospective cohort study. Convenience sampling was used in 11 studies (Brooks and Clark, 2019, A. King et al., 2020, D. King et al., 2020, Li et al., 2019, Macey and Hamari, 2019, Zendle, 2019, Zendle and Cairns, 2018, Zendle and Cairns, 2019, Zendle et al., 2020, Zendle et al., 2019), often recruiting participants through paid recruitment platforms such as Amazon Mechanical Turk or by posting in various forums such as Reddit. Two studies (Drummond et al., 2020, Zendle, 2020) used quota sampling, and one study (Kristiansen & Severin, 2020) employed random sampling and used a national database to select participants.

Six studies had their first author from the United Kingdom (Zendle, 2019, Zendle, 2020, Zendle and Cairns, 2018, Zendle and Cairns, 2019, Zendle et al., 2020, Zendle et al., 2019), two studies from Canada (Brooks & Clark, 2019), two from the United States (A. King et al., 2020, Li et al., 2019), and one from each Aotearoa New Zealand (Drummond et al., 2020), Australia (D. King et al., 2020), Denmark (Kristiansen & Severin, 2020), and Finland (Macey & Hamari, 2019). Please see Table 2 for more information regarding study design and characteristics.

**Table 2:** Study design and characteristics.

Study	Country‡	Study Design	Sampling Technique	Type of MT	Microtransaction Engagement Assessment	IGD Assessment tool	IGD Prev (%)	GambD Assessm-ent tool	GambD Prev (%)
Brooks and Clark (2019)†	Canada	Cross sectional	Convenience	LB	Opened a loot box? Have bought a loot box or ‘key’? Sold loot box or LB item?	IGDS-9 (Lemmens et al., 2015)	N/A*	PGSI (Ferris & Wynne, 2001)	7.6
Brooks and Clark (2019)†	Canada	Cross sectional	Convenience	LB	Opened a loot box? Have bought a loot box or ‘key’? Sold loot box or LB item?	IGDS-9 (Lemmens et al., 2015)	N/A*	PGSI (Ferris & Wynne, 2001)	2.6
Drummond et al. (2020)	NZ	Cross sectional	Quota	LB	RLI (Brooks & Clark, 2019)	Internet Gaming Disorder Checklist (adapted by researchers; Przybylski et al., 2016)	17	PGSI (Ferris & Wynne, 2001)	17
A. King et al., 2020;	USA	Cross sectional	Convenience	LB + NRMT	RLI (Brooks & Clark, 2019) adapted by authors	Clinical Assessment Tool (van Rooij et al., 2017)	23.6	SOGS-RA (Winters et al., 1993)	14.4
	Australia	Cross sectional	Convenience	LB + NRMT	Expenditure	Petry et al.'s (2014) Internet Gaming Disorder Criteria	14	NA	N/A
Kristiansen and Severin (2020)	Denmark	Cross sectional	Random sample	LB	Obtained a loot box? purchased a loot box or a key? S elling virtual items from loot box?	NA	N/A	SOGS-RA (Winters et al., 1993)	2.2
Li et al. (2019)	USA	Cross sectional	Convenience	LB	Purchased a loot box?	Restated DSM-V Criteria as questions – Author developed	20.06	PGSI (Ferris & Wynne, 2001)	48.55
Macey and Hamari (2019)	Finland	Cross sectional	Convenience	LB	Purchased a loot box?	N/A	N/A	PGSI (Ferris & Wynne, 2001)	4.5
Zendle (2019)	UK	Prospective Cohort Study	Convenience	All MT	Expenditure	N/A	N/A	PGSI (Ferris & Wynne, 2001)	N/A
Zendle (2020)	UK	Cross sectional	Quota	LB	Spent money on loot box in past 12 months?	IGDS-9 (Lemmens et al., 2015)	7.9%	PGSI (Ferris & Wynne, 2001)	2.4
Zendle and Cairns (2018)	UK	Cross sectional	Convenience	LB + NRMT	Expenditure	NA	N/A	PGSI (Ferris & Wynne, 2001)	1.4
Zendle and Cairns (2019)	UK	Cross sectional	Convenience	LB + NRMT	Expenditure	NA	N/A	PGSI (Ferris & Wynne, 2001)	17.7
Zendle et al. (2020)	UK	Cross sectional	Convenience	LB	Expenditure	NA	N/A	PGSI (Ferris & Wynne, 2001)	17.7
Zendle et al. (2019)	UK	Cross sectional	Convenience	LB + NRMT	Paid for loot boxes? Used loot box but not paid for loot box?	NA	N/A	CAGI (Tremblay et al., 2010)	15.7

LB = Loot Boxes. MT = Microtransactions. IGD = Gaming Disorder. GambD = Gambling Disorder. Prev = Prevalence. NRMT = Non-Random Microtransactions. All MT = All Microtransactions. IGDS-9 = Internet Gaming Disorder Scale – Short Form. PGSI = Problem Gambling Severity Index. SOGS-RA = South Oaks Gambling Screen – Revised for Adolescents. CAGI = Canadian Adolescent Gambling Inventory. NZ = New Zealand. UK = United Kingdom. USA = United States of America. ‡: Based on the first author’s affiliation. All studies with more than two authors have been written as ‘et al.’. **†**: Both entries pertain to the same publication, however they report to two separate studies.

### 3.4 Measurement and psychometric assessment

All studies investigated loot boxes, with five studies also investigating non-random microtransactions in addition to loot boxes (A. King et al., 2020, D. King et al., 2020, Zendle and Cairns, 2018, Zendle and Cairns, 2019, Zendle et al., 2019), and one study investigated all types of microtransactions (Zendle, 2019). Seven studies (Brooks and Clark, 2019, Kristiansen and Severin, 2020, Li et al., 2019, Macey and Hamari, 2019, Zendle, 2020, Zendle et al., 2019) measured microtransaction engagement by asking various questions regarding loot box engagement, five studies (D. King et al., 2020, Zendle, 2019, Zendle and Cairns, 2018, Zendle and Cairns, 2019, Zendle et al., 2020) considered expenditure as a measure of engagement, and two studies (Drummond et al., 2020, A. King et al., 2020) used the Risky Loot Box Index (RLI; Brooks & Clark, 2019).

The Internet Gaming Disorder Scale – 9 item scale (IGDS-9; Lemmens, Valkenburg, & Gentile, 2015) was used to assess for IGD in three studies (Brooks and Clark, 2019, Zendle, 2020). An adapted version of the Internet Gaming Disorder Checklist (Przybylski, Weinstein, & Murayama, 2016) was used in one study (Drummond et al., 2020), the Clinical Assessment Tool (C-VAT 2.0; van Rooij, Schoenmakers, & van de Mheen, 2017) was used in one study (A. King et al., 2020), Petry et al.'s (2014) Internet Gaming Disorder Criteria were used in one study (King, Russell, Delfabbro, & Polisena, 2020), and restating the DSM-5 criteria as questions was used in one study (Li et al., 2019). IGD was found to have a varied range of prevalence rates, ranging from 7.90% − 23.60%.

The Problem Gambling Severity Index (PGSI; Ferris & Wynne, 2001) was the most common tool for assessing gambling disorder and was used in 10 studies (Brooks and Clark, 2019, Drummond et al., 2020, Li et al., 2019, Macey and Hamari, 2019, Zendle, 2019, Zendle, 2020, Zendle and Cairns, 2018, Zendle and Cairns, 2019, Zendle et al., 2020). The South Oaks Gambling Screen – Revised for Adolescents (SOGS-RA; Winters, Stinchfield, & Fulkerson, 1993) was used in two studies (A. King et al., 2020, Kristiansen and Severin, 2020) and The Canadian Adolescent Gambling Inventory (CAGI; Tremblay, Stinchfield, Wiebe, & Wynne, 2010) was used in one study (Zendle et al., 2019). Prevalence rates of gambling disorder reported were variable, ranging from 1.40% to 48.55%.

### 3.5 Microtransactions and gambling disorder

A total of seven studies investigated the relationship between microtransactions and gambling disorder alone (Kristiansen and Severin, 2020, Macey and Hamari, 2019, Zendle, 2019, Zendle and Cairns, 2018, Zendle and Cairns, 2019, Zendle et al., 2020, Zendle et al., 2019). Those which included correlational analyses (Macey and Hamari, 2019, Zendle and Cairns, 2019, Zendle et al., 2020, Zendle et al., 2019) all revealed significant and positive correlations between loot box expenditure and gambling disorder severity, ranging from .17 to .35. One study (Zendle & Cairns, 2019) investigated microtransactions and found loot box expenditure to be positively correlated with other microtransaction expenditure (*ρ* = .45), and that microtransaction expenditure positively correlated with gambling disorder severity (*ρ* = .16). Loot box expenditure was more strongly and positively associated with gambling disorder severity (*ρ* = .24) than other microtransaction expenditure in this study (see Table 3). Again, loot box expenditure refers to payment allowing to open loot boxes, whereas microtransaction expenditures may include loot box expenditure and other in-game purchases providing customisation of in-game experiences.

**Table 3:** Correlation Coefficients for studies only including gambling disorder.

Study	Coefficient	LBSpend*GambD	LBSpend*MTSpend	MTSpend*GambD
Kristiansen and Severin (2020)	N/A*	N/A*	N/A*	N/A*
Macey and Hamari (2019)	τ	.17***	N/A	N/A
Zendle (2019)	N/A	N/A	N/A	N/A
Zendle and Cairns (2018)	N/A	N/A	N/A	N/A
Zendle and Cairns (2019)	ρ	.24***	.45***	.16***
Zendle et al. (2020)	ρ	.30***	N/A	N/A
Zendle et al. (2019)	ρ	.35***	N/A	N/A

LBSpend = Loot Box Expenditure. GambD = Gambling Disorder. MTSpend = Microtransaction Expenditure. N/A* = Author contacted for information and did not provide it. * = *p* < .05. ** = *p* < .01. *** = *p* < .001. τ = Kendall rank correlation coefficient. ρ = Spearman’s Rank Order Correlation coefficient. Values have been rounded to 2 decimal places. All studies with more than two authors have been written as ‘et al.’.

Five studies conducted mean difference analyses (Zendle, 2019, Zendle and Cairns, 2018, Zendle and Cairns, 2019, Zendle et al., 2020, Zendle et al., 2019) (see Table 4, Table 5). Of these, three studies had loot box expenditure as the dependent variable and problem gambling severity as the independent variable (Zendle and Cairns, 2018, Zendle and Cairns, 2019, Zendle et al., 2020). All three of these studies found a significant main effect (*p* < .001) of problem gambling severity on loot box expenditure, with effect sizes ranging from *η*^2^ = .05 to .60. Two of these same studies (Zendle and Cairns, 2018, Zendle and Cairns, 2019) conducted additional analyses with microtransaction expenditure as the dependent variable and problem gambling severity as the independent variable. Both found a significant main effect (*p* < .001) of problem gambling severity on loot box expenditure with effect sizes (*η*^2^) ranging from < .01 to .03.

**Table 4:** Mean loot box/microtransaction expenditure in studies only including gambling disorder.

Study	Type of Expenditure	M Expenditure Overall (SD)	M Expenditure NPGamb (SD)	M Expenditure LRGamb (SD)	M Expenditure MRGamb (SD)	M Expenditure ProbGamb (SD)
Kristiansen and Severin (2020)	N/A*	N/A*	N/A*	N/A*	N/A*	N/A*
Macey and Hamari (2019)	In-game expenditure	4.90 (16.35)	3.74 (8.32)	4.42 (9.29)	5.63 (16.08)	17.35 (59.35)
Zendle, 2019**†**	In-game expenditure	N/A	8.47 (N/A)	11.37 (N/A)	21.11 (N/A)	83.86 (N/A)
Zendle and Cairns (2018)	LB expenditure	N/A	N/A	N/A	N/A	N/A
Zendle and Cairns (2019)	LB expenditure	19.58 (N/A)	11.14 (N/A)	21.87 (N/A)	27.55 (N/A)	38.24 (N/A)
	Other MT expenditure	44.32 (N/A)	40.12 (N/A)	30.98 (N/A)	36.07 (N/A)	78.83 (N/A)
Zendle et al. (2020)	N/A	N/A	N/A	N/A	N/A	N/A
Zendle et al. (2019)	LB Expenditure	N/A*	3.64	6.16		21.3

M = Mean. SD = Standard Deviation. LB = Loot box. MT = Microtransaction. NPGamb = Non-problem Gamblers. LRGamb = Low-risk Gamblers. MRGamb = Moderate-risk Gamblers. ProbGamb = Problem Gamblers. Where LRGamb and MRGamb are combined they were classified in the article as ‘at risk gamblers’. N/A* = Author contacted for information and did not provide it. All values are in United States Dollar. Values have been rounded to 2 decimal places. **†** = Expenditure values reported are before loot boxes were removed from the game. Values for after loot boxes were removed are available in the original publication. All studies with more than two authors have been written as ‘et al.’.

**Table 5:** Mean difference analyses for studies only including gambling disorder.

	Mean Difference Estimates				Pairwise Analysis						
Study	Analysis	DV	IV	Main Effect (Effect size)	Alpha	NPGamb*LR Gamb	NPGamb*MR Gamb	NPGamb*PGamb	LRGamb*MRGamb	LRGamb*PGamb	MRGamb*PGamb
Kristiansen and Severin (2020)	N/A	N/A	N/A	N/A	N/A	N/A	N/A	N/A	N/A	N/A	N/A
Macey and Hamari (2019)	N/A	N/A	N/A	N/A	N/A	N/A	N/A	N/A	N/A	N/A	N/A
Zendle (2019)	4 × 2 mixed-design ANOVA	In-game Expenditure	PGamb Severity	$\eta_{\text{p}}^{\text{2}}$ = 0.19	N/A	N/A*	N/A*	N/A*	N/A*	N/A*	N/A*
Zendle and Cairns (2018)	Kruskal Wallis H Test	LB expenditure	PGamb Severity	*χ^2^* (3) = 284.26, *p* < .001* (*η*^2^ = .05)	.008	*p* < .001*	*p* < .001*	*p* < .001*	*p* < .001*	*p* < .001*	*p < .* 002*
		Other MT expenditure	PGamb Severity	*χ^2^* (3) = 38.62, *p* < .001* (*η*^2^ < .01)	N/A	*p* < .001*	*p* < .001*	*p* = .072	*p* < .001*	*p = .*517	*p* = .166
Zendle and Cairns (2019)	Kruskal Wallis H Test	LB Expenditure	PGamb Severity	*χ^2^* (3) = 62.85, *p* < .001*, (*η*^2^ = .05)	.008	*p < .*001*	*p < .*001*	*p < .*001*	*p < .*416	*p < .*163	*p < .*932
		Other MT expenditure	PGamb Severity	*χ^2^* (3) = 32.47, *p* < .001*, (*η*^2^ = .03)	.008	*p < .*001*	*p < .*001*	*p* < .001*	*p* = .099	*p* = .642	*p* = .243
Zendle et al. (2020)	Mann-Whitney *U* test	PGambSeverity	Pays for LB (Yes or No)	*U* = 122117, *p* < .001* (*η*^2^ = .60)	N/A	N/A	N/A	N/A	N/A	N/A	N/A
Zendle et al. (2019)	Kruskal Wallis H Test	LB Expenditure	PGamb Severity	*x^2^* (2) = 108.48, *p* < .001* (*η*^2^ = .12)	.0041	*p < .*001*		*p < .*001*	NA	*p* < .001*	

*Note:* The information presented was extracted from published studies that were eligible for review. *Abbreviations*: NPGamb = Non-problem Gamblers. LRGamb = Low-risk Gamblers. MRGamb = Moderate-risk Gamblers. ProbGamb = Problem Gamblers. LRGamb and MRGamb were sometimes combined as ‘at risk problem gambling’. N/A* = Author contacted for information and did not provide it. *p* values marked with * were significant. Zendle (2019) found an interaction effect which is available in the original study. All studies with more than two authors have been written as ‘et al.’.. Main effect statistics have been rounded to 2 decimal places.

Furthermore, Zendle (2019) used overall in-game expenditure as the dependent variable and problem gambling severity as the independent variable in relation to one specific video game (i.e., Heroes of the Storm). The results of this study reported a significant main effect (*p* < .001) of problem gambling severity on in-game expenditure with an effect size of $\eta_{\text{p}}^{\text{2}}$ = .19 and found that problem gamblers spent significantly less money (U.S. dollars; USD) in-game after the loot boxes were removed from the game. Zendle et al. (2020) had problem gambling severity as the dependent variable and whether a person pays for loot boxes (i.e., dichotomous: yes or no) as the independent variable. The results revealed a significant main effect of paying for loot boxes on problem gambling severity (*p* < .001), with an effect size of *η*^2^ = .60 (see Table 5).

Table 5 reports all pairwise analyses of the three studies (Zendle and Cairns, 2018, Zendle and Cairns, 2019, Zendle et al., 2019) that used loot box expenditure as the dependent variable and problem gambling severity as the independent variable. Results revealed that individuals in the non-problem gambling group spent significantly less money (USD) on loot boxes than individuals at risk of problem gambling (i.e., low-risk and moderate-risk gamblers) and individuals who meet criteria for problem gambling (*p < .*001). Two studies (Zendle and Cairns, 2018, Zendle et al., 2019) also found that individuals at risk of problem gambling spent significantly (USD) less money on loot boxes than individuals who met criteria for problem gambling (*p < .*001–.002), however these results were not replicated in the Zendle and Cairns (2019) study.

Two studies (Zendle and Cairns, 2018, Zendle and Cairns, 2019) included pairwise analyses with other microtransaction expenditure as the dependent variable and problem gambling severity as the independent variable. Both studies found that non-problem gamblers spent significantly less money than those at risk of problem gambling and those who meet criteria for problem gambling (*p < .*001). Zendle and Cairns (2018) also found that individuals with low-risk of problem gambling spent significantly less than those at moderate-risk of problem gambling.

### 3.6 Microtransactions and IGD

Only one study (King, Russell, Delfabbro, & Polisena, 2020) examined the relationship between various microtransactions and IGD alone. This study conducted a logistical regression analysis and reported unstandardised coefficients. As these cannot be compared to the other studies conducting similar regression analyses that reported standardised coefficients, they were not extracted. Further, this study assessed loot box expenditure within a single game (i.e., Fortnite) and its association with IGD symptoms and did not find a statistically significant association.

### 3.7 Microtransactions, IGD, and gambling disorder

Six studies investigated the relationship between microtransactions and both IGD and gambling disorder (Brooks and Clark, 2019, Drummond et al., 2020, A. King et al., 2020, Li et al., 2019, Zendle et al., 2020). Four studies examined the association between IGD and gambling disorder (Brooks and Clark, 2019, Drummond et al., 2020, A. King et al., 2020) and reported positive correlations ranging from .19 to .60. The same four studies also found positive correlations between the RLI and IGD, ranging from .32 to .60, and the RLI and gambling disorder, ranging from .32 to .49.

Three studies investigated the association between the RLI and loot box expenditure (Brooks and Clark, 2019, Drummond et al., 2020) and reported positive correlations ranging from .25 to .49. Three studies investigated the association between loot box expenditure and IGD (Brooks and Clark, 2019, Zendle, 2020). Two of these found a positive correlation of .18 and .41 (Brooks and Clark, 2019, Zendle, 2020), however Brooks and Clark's (2019) second study did not find a significant association between loot box expenditure and IGD.

Four studies examined the relationship between loot box expenditure and gambling disorder (Brooks and Clark, 2019, Drummond et al., 2020, Zendle, 2020). Three of these (Brooks and Clark, 2019, Drummond et al., 2020, Zendle, 2020) found positive correlations ranging from .21 to .34, however Brooks and Clark's (2019) second study did not find a significant relationship between loot box expenditure and gambling disorder. Moreover, one study (A. King et al., 2020) found positive relationships between all microtransaction expenditure and both IGD and gambling disorder levels (.46 and .47, respectively), suggesting that higher expenditure is associated with greater symptoms of IGD and gambling disorder (see Table 6).

**Table 6:** Correlation Coefficients for studies including both gaming disorder and gambling disorder.

Study	Coefficient	IGD*GambD	LBSpend*IGD	LBSpend*GambD	MTSpend*IGD	MTSpend*GambD	RLI*IGD	RLI*GambD	RLI*LBSpend
Brooks and Clark (2019)†	*r*	.43**	.18*	.23*	N/A	N/A	.36**	.49**	.49**
Brooks and Clark (2019)†	*r*	.19*	< -.01	< -.01	N/A	N/A	.32**	.32**	.25**
Drummond et al. (2020)	*ρ*	.60***	N/A	.34***	N/A	N/A	.60***	.41**	.39***
King, Wong-Padoongpatt et al. (2020)	*r*	.38**	N/A	N/A	.46**	.47***	.53***	.46**	N/A
Zendle (2020)	*ρ*	NR	.41***	.21***	N/A	N/A	N/A	N/A	N/A

IGD = Gaming Disorder. GambD = Gambling Disorder. LBSpend = Loot Box Expenditure. MTSpend = Microtransaction Expenditure. RLI = Risky Loot Box Index. * = *p* < .05, ** = *p* < .01, *** = *p* < .001. *r =* Pearson’s Correlation Coefficient. *ρ* = Spearman’s Rank Order Correlation coefficient. All studies with more than two authors have been written as ‘et al.’. Values have been rounded to 2 decimal places. **†**: Both entries pertain to the same publication, however they report to two separate studies.

One study (Drummond et al., 2020) examined loot box expenditure and conducted pairwise comparisons investigating the difference in loot box expenditure between problem gambling groups. The overall mean expenditure was 12.92 USD (SD = 23.29). The non-problem gambling group had the lowest average expenditure (M = .88 USD, SD = 5.24), followed by low-risk gamblers (M = 1.88 USD, SD = 7.82), which was followed by the moderate-risk gamblers (M = 4.64 USD, SD = 15.60), with problem gamblers having the highest expenditure (M = 12.91 USD, SD = 23.29). Simple effects analysis revealed significant differences in expenditure between all groups of gamblers, except for non-problem gamblers and low-risk gamblers (see Table 7).

**Table 7:** Mean difference analysis for Drummond et al. (2020).

M-Diff Estimates				Pairwise Analysis						
Analysis	DV	IV	Main Effect (Effect size)	Alpha	NPGamb*LR Gamb	NPGamb*MR Gamb	NPGamb*PGamb	LRGamb*MRGamb	LRGamb*PGamb	MRGamb*PGamb
One-way ANOVA	LB Expenditure	GambD Classifi-cation	*F*(3, 1045) = 48.50 *p* < .001 (N/A)	N/A	*p* = .553.	*p* < .001***	*p* < .001***	*p* = .017*	*p* < .001***	*p* < .001***

DV = Dependent Variable. IV = Independent Variable. NPGamb = Non-problem Gamblers. LRGamb = Low-risk Gamblers. MRGamb = Moderate-risk Gamblers. ProbGamb = Problem Gamblers. LB = Loot box. GambD = Gambling Disorder. *p* values marked with * were significant. ANOVA = Analysis of Variance. *F* statistic has been rounded to 2 decimal places.

Four studies conducted regression analyses and each study used different outcome variables (Brooks and Clark, 2019, Drummond et al., 2020, Li et al., 2019, Zendle, 2020). The results of these studies revealed that IGD was found to significantly predict RLI scores, loot box expenditure, and loot box purchasing behaviours. Additionally, gambling disorder was found to significantly predict loot box expenditure and loot box expenditure was found to predict both IGD and gambling disorder symptoms (see Table 8).

**Table 8:** Regression coefficients for studies including both gaming disorder and gambling disorder.

		Standardised Regression Coefficient (CI/SE)		
Study	Outcome Variable	IGD	GambD	LB Expenditure
Brooks and Clark (2019)	RLI	.19** (.06–.32/.07)	.17 (−.03–.35/.09)	N/A
Drummond et al. (2020)	LB Expenditure	.19***(N/A)	.27***(N/A)	N/A
Li et al. (2019)	LB Purchasing Behaviours	.22*** (N/A/.05)	.14*** (N/A/.04)	N/A
Zendle (2020)	IGD	N/A	N/A	.69***(NR/.12)
	GambD	N/A	N/A	.31***(NR/.09)

CI = Confidence Interval. SE = Standard Error. IGD = Gaming Disorder. GambD = Gambling Disorder. LB = Loot Box. RLI = Risky Loot Box Index. * = *p* < .05, ** = *p* < .01, *** = *p* < .001. All studies with more than two authors have been written as ‘et al.’. Values have been rounded to 2 decimal places.

### 3.8 Quality appraisal

The mean percentage on the AXIS tool was 71.22% (range 55.56%–83.33%) falling in the ‘Good’ category. Out of the 14 studies assessed, no studies fell in the ‘Poor’ or ‘Fair’ categories, nine studies fell in the ‘Good’ category, and five studies fell in the ‘Excellent’ category. In reference to the individual AXIS questions, there were specific questions that were generally not answered as ‘Yes’ across the body of the evidence. Two studies (Drummond et al., 2020, Zendle et al., 2020) received ‘Yes’ scores for question 3 ('Was the sample size justified?'), question 6 ('Was the selection process likely to select subjects/participants that were representative of the target/reference population under investigation?') was only assessed as ‘Yes’ on three studies (Drummond et al., 2020, Kristiansen and Severin, 2020, Zendle, 2020), question 7 ('Were measures undertaken to address and categorise non-responders?') was scored as ‘Yes’ for three studies (D. King et al., 2020, Macey and Hamari, 2019, Zendle, 2020), and question 10 ('Is it clear what was used to determined statistical significance and/or precision estimates? (e.g. p-values, confidence intervals)') was only assessed as ‘Yes’ on six studies (Brooks and Clark, 2019, A. King et al., 2020, D. King et al., 2020, Macey and Hamari, 2019, Zendle, 2020). Additionally, question 13 ('Does the response rate raise concerns about non-response bias?') could only be assessed at all for three studies with two ‘Yes’ scores (D. King et al., 2020, Kristiansen and Severin, 2020), and question 14 ('If appropriate, was information about non-responders described?') could only be assessed on two studies with one ‘Yes’ score (King, Russell, Delfabbro, & Polisena, 2020) (see Table 9).

**Table 9:** Appraisal tool for Cross-Sectional Studies (AXIS) Quality Appraisal Assessment.

AXIS = Appraisal Tool for Cross-sectional Studies. Green squares represent a rating classed as ‘Yes’ in relation to the criterion assessed. Red squares represent a rating classed as ‘No’ in relation to the criterion assessed. Yellow squares represent an rating that was 'Maybe' in relation to the criterion assessed. Grey squares represent the criterion in question was not assessable. Category classification thresholds: Poor = 0–24%, Fair = 25–49%, Good = 50–74%, Excellent = 75–100%

*: The wording of question 13 was slightly shifted for ease of reporting so that ‘Yes’ responses all indicated positive ratings. **†**: Both entries pertain to the same publication, however they report to two separate studies. All studies with more than two authors have been written as ‘et al.’.

## 4 Discussion

The present study encompassed a preregistered systematic review on the existing literature to clarify the relationship between microtransactions, IGD, and gambling disorder within the context of different types of in-game microtransactions. Across all studies that investigated only gambling disorder, a significant and positive relationship was found between greater severity of gambling disorder symptoms and increased in-game microtransaction expenditure. Additionally, the studies reviewed found that the amount of money individuals spend on microtransactions increased as the risk of gambling disorder also increased. Moreover, the positive relationship found between loot boxes and gambling disorder was stronger than for other types of microtransactions (e.g., *ρ* = .24 vs *ρ* = .16) in one study (Zendle & Cairns, 2019), while another study (Zendle, 2019) reported that in-game expenditure (in Heroes of the Storm) decreased after loot boxes were removed from the game. These results support the positive link between gambling disorder and loot boxes specifically. The nature of loot boxes, which is akin to gambling (McCaffrey, 2019), may be underlying the relationship between microtransaction expenditure and gambling disorder symptoms.

Furthermore, only one study (King, Russell, Delfabbro, & Polisena, 2020) investigated microtransactions and IGD in isolation. This study only assessed these variables in a single game (i.e., Fortnite) and did not find a significant relationship between microtransaction expenditure on Fortnite and IGD. It is important to note that given its narrow focus, the findings reported in this study may not be generalisable to the broader microtransaction context in other video games.

The studies where microtransactions and both IGD and gambling disorder were investigated simultaneously found significant positive relationships between IGD and gambling disorder. Additionally, significant relationships were found between loot box expenditure with both IGD and gambling disorder. Significant positive relationships were also identified between microtransaction expenditure and both IGD and gambling disorder. However, Brooks and Clark's (2019) second study was the only study that did not identify a significant relationship between loot box expenditure with IGD and gambling disorder. There was also a significant positive relationship between the RLI and IGD, gambling disorder, and loot box expenditure. Drummond et al. (2020) found that the amount individuals spent on loot boxes increased as the risk of gambling disorder also increased. Furthermore, IGD was also found to significantly predict greater RLI scores and loot box expenditure, and loot box expenditure significantly predicted higher levels of IGD and problem gambling severity.

The positive correlation between microtransaction expenditure and gambling disorder severity may be due to the fact that certain types of microtransaction engagement present with gambling-like features, particularly when it comes to loot boxes (Drummond & Sauer, 2018). Moreover, modern video games feature other gambling-like practices that are associated with both gaming and gambling disorder (Zendle et al., 2020), while additional literature has reported that paying for loot boxes is also linked to problem gambling (Zendle et al., 2020).

Thus, there is overall convincing evidence for a positive relationship between microtransaction expenditure with both IGD and gambling disorder. Specifically, this relationship appears to be stronger with loot boxes compared to other non-random microtransactions. However, the way an individual engages with loot boxes may contribute to this relationship. Risky loot box use was found in multiple studies to be positively associated with both gambling disorder, IGD, and loot box expenditure. It is plausible that risky loot box usage may mediate the relationship between microtransactions and IGD and/or gambling disorder.

While it is evident that there is a positive relationship between microtransaction expenditure with both IGD and gambling disorder, it is important to also understand the impact that this relationship may have on the lives of individuals. Carey, Delfabbro, and King (2021) identified that IGD was associated with higher levels of general harm to an individual and were six times more likely to experience risk of both physical harm (e.g., poor sleep, diet, hygiene, and drug use), psychological harm (e.g., anxiety, depressed, or general psychological distress), and higher levels of financial harm. Interestingly, a recent review on the characteristics of gamers who purchase loot boxes (Yokomitsu et al., 2021) identified that loot box expenditure was associated with both positive mood, psychological distress, and negative mood. The association with positive mood, negative mood, and psychological distress may appear counter intuitive but plausible nevertheless if considered within an engagement process. Firstly, positive mood could emerge as an anticipatory response to the purchasing and potential reward associated with the loot box (i.e., high value reward). Following this, negative mood and psychological distress could ensue, particularly in cases where the reward did not present high psychological and in-game value (i.e., low value reward).

Moreover, Drummond et al. (2020) suggested the relationship with positive mood could be due to the benefits of purchasing items that are congruent with a person’s personality, and that gamers with disposable income to be spent on loot boxes may be experiencing a benefit to their mood. Additionally, they argued that the act of opening loot boxes can be ‘fun’, much like traditional gambling, and that a relationship with positive mood does not take away from the associated risk of financial harm.

Two studies (Kristiansen and Severin, 2020, Zendle et al., 2019) investigated only adolescent samples. Zendle et al. (2019) conducted correlation analyses and identified a significant positive correlation between loot box spending and gambling disorder. Interestingly, this relationship was stronger than in other studies that included adult samples (Brooks and Clark, 2019, Zendle, 2020, Zendle and Cairns, 2019, Zendle et al., 2020). One study (Drummond et al., 2020) that investigated the general population also found a positive relationship of similar strength to Zendle et al. (2019) between loot box spending and gambling disorder, whereas another study (Macey & Hamari, 2019) that included both adolescents and adults found a weaker positive relationship between loot box spending and gambling disorder. However, neither of these studies included a comparison between adolescent and adults.

These findings may suggest that adolescents who purchase loot boxes are at a higher risk of developing gambling disorder symptoms. This is unsurprising considering adolescents have poorer impulse control and decision making due to their developing prefrontal cortex and subcortical limbic regions (Casey, Getz, & Galvan, 2008). Consequently, this can result in increased risk taking and negative outcomes (Casey et al., 2008). Gambling disorder is heavily associated with deficits in impulse control (Slutske, Caspi, Moffitt, & Poulton, 2005) and therefore engaging with loot boxes in adolescence, when there is a natural lack of impulse control, may leave adolescents at a higher risk of developing gambling disorder symptoms than adults.

Therefore, the links between microtransactions such as loot boxes with IGD and gambling disorder, as well as their associated risk of harm to an individual, have significant implications for policy-making decisions. Loot boxes remain largely unregulated, with the exception of some European nations (McCaffrey, 2019). While causality cannot be established in this relationship due to the cross-sectional nature of the existing evidence, future research should further explore the relationships identified in the present study in order to ascertain the need for regulatory actions within the industry. This is a key aspect to be explored, particularly amongst minors since a recent study (González-Cabrera et al., 2022) found that individuals under the age of 18 engaged in loot box purchasing behaviours to the same extent as adults.

Consequently, this highlights the importance of protecting children and adolescents who engage in both video gaming and loot box activities regularly from potential harms. For instance, the Parliament of Australia has identified that loot boxes fall into a legal grey area as to whether they are constituted as gambling, however has pushed for further regulations and inquiry (Australian Parliament House, 2018). It was identified that video games with loot boxes psychologically resembling gambling should be age-restricted as either MA15 + or R18 + and labelled with a content descriptor of ‘Simulated Gambling’. Additionally, they call for clear disclosure of the odds of loot box outcomes and the development of an ethical framework to guide video game production. However, such recommendations are yet to be implemented. The introduction of policies such as these, in cooperation with video game production companies, would help protect vulnerable individuals from loot box related harms and are further supported by the findings of this study. In relation to the ethical design of video games, Montag et al. (2019) called for social responsibility (as recently also mentioned in the context of social media; see Montag, Hegelich, Sindermann, Rozgonjuk, & Marengo, 2021) as it is of utmost importance.

Overall, the evidence quality of the studies reviewed was deemed as being in the ‘Good’ category. A closer analysis of the questions that were adopted to assess the studies reviewed showed some common methodological issues that were consistent throughout many of the studies. Most studies used convenience sampling, which resulted in very few studies having samples that represented the target population, thus limiting the external validity of the body of evidence reviewed. Additionally, as is the nature of online survey studies, many studies were unable to address or categorise non-responders in their survey. This made it impossible for the most part to rule out non-response bias as contributing to the results found in most studies.

Furthermore, only six studies met the AXIS criterion for having clarity in terms of the significance and/or precision estimates reported. This required studies to explicitly state the statistical methods, software packages, and significance levels that were used. Most commonly studies did not state what α level was used to ascertain statistical significance or the statistical package utilised. While it is likely that the α levels were implied to be .05, this was not made explicit in the reviewed studies. Additionally, only two studies justified their sample sizes, making it difficult to interpret the results meaningfully and limits the quality of the evidence.

Interestingly, the quality assessment conducted in this review varied to some extent from other reviews (Spicer et al., 2021, Yokomitsu et al., 2021), with the current review ratings being higher. Spicer et al.'s (2021) review included many older studies published prior to 2013, and stated that the quality of the evidence on loot box papers has increased in more recent years. This may explain some of the difference in quality appraisal between their assessment and the current review as only newer studies published after 2013 were included. However, it is unclear why there is such a difference between Yokomitsu et al.'s (2021) appraisal and the current one considering the approach to assessment was very similar. It is possible that the difference in quality assessment may be caused by using different assessment measures or due to difference in rater opinions. Neither review included an inter-rater assessment and therefore it is unclear how much variance in opinion attributed to the quality ratings.

When examining the characteristics of participants, the majority of studies were dominated by Caucasian individuals mostly from North America, the United Kingdom, Australia, and other countries in Europe. Additionally, most samples had a majority of male participants. This is unsurprising considering that only two studies had representative samples. No studies included in this review targeted an exclusive Asian population, and only one study (Brooks & Clark, 2019) had a high number of participants who described themselves as Asian. This is concerning considering the fact that the prevalence of both IGD and gambling disorder is higher in Asia than Europe and the USA (Montag and Becker, 2020, Stevens et al., 2021, Abbott, 2017).

In reference to the general design and study characteristics, all studies but one (Zendle, 2019) were cross-sectional. Prevalence rates of IGD varied drastically between studies and were higher than the prevalence rates recently reported by Stevens et al. (2021), which ranged from 1.96% to 3.05%. The IGDS-9 was the most used tool to assess for IGD. It is possible that the discrepancy in prevalence rates may be due to non-representative samples and selection bias stemming from the recruitment of convenience and self-selected samples. However, five different methods of assessment were used across seven studies which may also have contributed to the observed heterogeneity in prevalence rates reported among the reviewed studies as this is a known issue likely to bias prevalence estimates (Stevens et al., 2021).

The way microtransaction engagement was measured across studies was also variable, and there was little consistency between studies in how this was assessed. Often these were researcher developed *ad hoc* questions about whether an individual had obtained a loot box, used a loot box, bought a loot box or loot box key, or sold virtual items they obtained from a loot box. Loot box and microtransaction expenditure and the RLI were also used to measure microtransaction engagement. One study (A. King et al., 2020) adapted the RLI and two studies (Macey and Hamari, 2019, Zendle, 2019) converted their questions on engagement into constructs of engagement and were not piloted previously.

It is evident that there is little consistency among researchers about what tools should be used in the assessment of IGD and microtransaction engagement. A recent study has developed a promising tool to measure microtransaction engagement (the Problematic Use of Loot Boxes Questionnaire; PU-LB; González-Cabrera et al., 2022) in a Spanish sample to assess factors that underly loot box purchasing behaviours. This work demonstrated sound psychometric properties and may prove useful in further research to better understand what motivates loot box usage.

Gambling disorder prevalence rates also varied significantly between studies and were different to general prevalence rates, ranging from .1% to 6.0% (Abbott, 2017). However, gambling disorder was more consistently assessed across studies when compared to IGD and microtransaction engagement. The PGSI was used in most studies reviewed, with the SOGS-RA and CAGI being appropriately chosen for adolescent populations. Nevertheless, it is likely that the variance in prevalence rates is also attributed to the non-representative samples and selection bias.

### 4.1 Limitations of the current review

The current review did not take into consideration other variables that may relate to microtransactions, IGD, and/or gambling disorder such as psychological distress or impulsivity. Therefore, the role of these variables has not been accounted for in the present review.

Additionally, the database search was conducted in September of 2020. While it would have been optimal to re-run these searches and integrate newly published studies into the review, this was not possible due to time constraints. Therefore, it is possible that new data is available on this topic which has not been included in this review. Thus, interpretation of the findings reported must take the search date into consideration.

### 4.2 Limitations of the evidence

The body of evidence reviewed in this paper is largely correlational and therefore causality has not been robustly established regarding the relationships between microtransactions, risky loot box use, IGD, and gambling disorder. Additionally, the sampling methods employed have resulted in non-representative samples across most studies and impacts the external validity of the evidence. Methods of assessing IGD and microtransaction engagement were inconsistent across studies and may account for some of the variance in the evidence. It is important to acknowledge that the validity of the methods used to examine such rapidly evolving technology may also need to be updated to enable greater levels of validity and reliability.

Furthermore, while the studies included assessed IGD using diagnostic or screening measures, no studies conducted clinical interviews. Therefore, the current research on this topic measures clinical symptomology and not clinically diagnosed samples.

### 4.3 Future directions

To support the push for policy development, implementation, and better regulation of loot boxes, it is paramount that future studies investigate causality within the relationships between microtransactions and both IGD and gambling disorder as this will encourage and guide policy-makers on what type of regulations may prove beneficial. Additionally, it is important that future research investigate the relationship between microtransactions with IGD and gambling disorder in other cultures to ensure that appropriate recommendations can be made regarding policy and intervention that are culturally and clinically sensitive rather than based solely on Western individualistic populations. Furthermore, researchers should adopt a more consistent and standardised method of assessment for both microtransaction engagement and IGD to ensure accurate measurement of these variables and improve the internal validity of the body of evidence. To this end, new standardised tools assessing disordered gaming according to the latest WHO framework (e.g., the Gaming Disorder Test; see Pontes, 2021) may prove beneficial.

To increase the quality of evidence of future studies on this topic, there should be a focus on using sampling methods which are more likely to yield a representative sample, using recruitment techniques that enable insight into non-responders and comparison between non-responders and responders, reporting all α levels and relevant statistical cut-offs as well as the software packages used, and justifying their sample size to ensure this growing body of research is robust and generalisable. Furthermore, using clinically diagnosed samples may provide a clearer picture on the relationships identified in the present review and improve the overall quality of the evidence. Additionally, it may be beneficial for future reviews to include an inter-rater assessment regarding the quality of the evidence to improve transparency and review quality.

Future reviews on this topic should synthesise the evidence on how variables such as psychological distress or impulsivity relate to microtransactions, IGD, and/or gambling disorder not only in a general and broader context but also within subgroups (e.g., male, female). This may provide invaluable information on factors that contribute to this relationship and may also help highlight potential targets for therapeutic intervention. As it is plausible that risky loot box usage may mediate the relationship between microtransactions and IGD and/or gambling disorder, future research should investigate the relationship between risky loot box use and its underlying cognitive distortions and behaviours. These may prove to be important targets for cognitive behavioural interventions (Dong and Potenza, 2014, Yokomitsu et al., 2021).

Future measuring of actual recorded behaviours and applying digital phenotyping principles may yield promising findings, but this clearly needs support from the industry (Montag & Rumpf, 2021).

## 5 Conclusion

In conclusion, this review identified a clear positive relationship between microtransactions with IGD and gambling disorder with evidence to suggest that the gambling-like nature of loot boxes may underlie this relationship. Additionally, there is evidence to suggest that the way an individual engages with loot boxes, such as risky loot box use, could mediate or moderate the relationship between microtransactions with IGD and gambling disorder. Furthermore, there is evidence to suggest that adolescents who purchase loot boxes may be at greater risk of developing gambling disorder. It is important to note that these findings are largely correlational in nature and that causality has not been established.

The current systematic review also identified a lack of generalisability and cross-cultural validity within the currently body of evidence due to sampling practices and the nature of online surveys resulting in very few representative samples. There is also a clear need for more consistency between researchers in how to assess both IGD and microtransaction engagement.

Based on these fundings, the current review calls for policies and industrial changes to be made regarding loot boxes in video games to protect vulnerable individuals from harm. These include MA15+ or R18+ age and ‘Simulated Gambling’ classifications for video games with loot boxes, transparency of the odds associated with loot box outcomes, and the development of an ethical framework to guide video game production (Australian Parliament House, 2018).

### Credit authorship contribution statement

**Phillip C. Raneri:** Conceptualization, Data curation, Formal analysis, Investigation, Methodology, Project administration, Validation, Visualization, Writing – original draft, Writing – review & editing. **Christian Montag:** Writing – review & editing, Visualization, Validation, Investigation. **Dmitri Rozgonjuk:** Writing – review & editing, Visualization, Validation, Investigation. **Jason Satel:** Writing – review & editing, Visualization, Validation, Investigation, Supervision. **Halley M. Pontes:** Conceptualization, Data curation, Formal analysis, Investigation, Methodology, Project administration, Validation, Visualization, Writing – original draft, Writing – review & editing, Supervision.

## Declaration of Competing Interest

The authors declare that they have no known competing financial interests or personal relationships that could have appeared to influence the work reported in this paper.

## Footnotes

1 While non-responding authors may have had different valid reasons for not responding to our email, we would like to emphasise that sharing of such research information is critical to improving the quality of research in general and increase replicability in Psychology.
